# Application of three-dimensional reconstruction technology in dentistry: a narrative review

**DOI:** 10.1186/s12903-023-03142-4

**Published:** 2023-09-04

**Authors:** Yueyan Cen, Xinyue Huang, Jialing Liu, Yichun Qin, Xinrui Wu, Shiyang Ye, Shufang Du, Wen Liao

**Affiliations:** State Key Laboratory of Oral Diseases & National Clinical Research Center for Oral Diseases, Department of Orthodontics, West China Hospital of Stomatology, Sichuan University, No.14, 3Rd Section of Ren Min Nan Rd. Chengdu, Sichuan, 610041 China

**Keywords:** Digital impression, Facial scanner, Intraoral scanner, Computerization

## Abstract

**Background:**

Three-dimensional(3D) reconstruction technology is a method of transforming real goals into mathematical models consistent with computer logic expressions and has been widely used in dentistry, but the lack of review and summary leads to confusion and misinterpretation of information. The purpose of this review is to provide the first comprehensive link and scientific analysis of 3D reconstruction technology and dentistry to bridge the information bias between these two disciplines.

**Methods:**

The IEEE Xplore and PubMed databases were used for rigorous searches based on specific inclusion and exclusion criteria, supplemented by Google Academic as a complementary tool to retrieve all literature up to February 2023. We conducted a narrative review focusing on the empirical findings of the application of 3D reconstruction technology to dentistry.

**Results:**

We classify the technologies applied to dentistry according to their principles and summarize the different characteristics of each category, as well as the different application scenarios determined by these characteristics of each technique. In addition, we indicate their development prospects and worthy research directions in the field of dentistry, from individual techniques to the overall discipline of 3D reconstruction technology, respectively.

**Conclusions:**

Researchers and clinicians should make different decisions on the choice of 3D reconstruction technology based on different objectives. The main trend in the future development of 3D reconstruction technology is the joint application of technology.

## Background

Medical imaging is known to play a critical role in disease diagnosis and treatment in all medical fields today [1], especially in dentistry, the surface contours inside or outside the mouth are the most visual and abundant kind of physical information received by the physician’s or the patient’s visual system, and they make relevant judgments based on this three-dimensional(3D) vision [2, 3]. Therefore, the development of dentistry is closely related to the trend of transitioning from two-dimensional(2D) images to 3D models of imaging targets in the field of computer vision [4].

3D reconstruction technology, one of the most popular research fields in computer vision technology, is a technology that explores equipping computers with eyes (transmitters) and brains (algorithms) to mimic the human visual system, involving multiple disciplinary systems including image processing, stereo vision, and more, to carve the real scene into a mathematical model that conforms to the computer's logical expression [1, 5-13]. Its strengths lie in 3D recording, visualization, reproduction, and reconstruction [14]. In recent years, 3D reconstruction technology has achieved great success and broader application through decades of development; both hardware and software have been greatly enhanced, and the imaging scale has become larger and with increased accuracy [15, 16].

The ability of 3D construction to acquire, reproduce, process, analyze, and understand static and dynamic images of dental procedures in real time [6] represents the ability to better describe or diagnose each disease and analyze it in further detail. When applied to imaging targets related to dental treatment, the advantages of 3D construction are mainly reflected in the following aspects:

1. 3D imaging has higher accuracy and more similarity to the actual shape of the scanned target [15, 17-21].
2. 3D imaging technology compensates for the general planar effect of a single perspective and brings a full range of feelings to doctors, patients, and researchers. Its abundant imaging results can be measured and analyzed at any angle and distance [15, 22], including the target object structure depth and other spatial information that two-dimensional images do not provide [18].
3. 3D imaging has strong communication utility. It can be manipulated by a doctor or patient to interactively observe the scan target from any angle. In addition, it makes communication more efficient and accurate, based on 3D images under remote conditions [18, 23, 24].
4. Both intraoral and extraoral 3D surface imaging data can be used to enrich and expand dentistry research. For example, literature [25-27] indicates that certain diseases can also be diagnosed using 3D surface imaging technology.
5. 3D imaging techniques reduce patient discomfort [28] and shorten the duration of the examination or treatment [21, 29-32], especially for patients who may have a vomiting reflex [33].
6. The time and operating steps are greatly reduced compared to traditional workflow [34-36], but at the same time, 3D imaging is easier to use [37-39].
7. All images can also be stored in digital form [18, 27] and are often paired with software programs developed to analyze 3D data accurately and reliably [40]. Easy access [41] greatly reduces the pressure on the physical storage space [35, 42, 43] and the ability to share information with other experts via the Internet [44].
8. It can avoid the deformation of the impression material and plaster model while ensuring the authenticity and accuracy of the scanner [35].

Based on the above, 3D reconstruction technology has been widely used for 3D imaging and analysis of orthodontic treatment, dental rows for restorative treatment, craniofacial bone, soft tissue, dental casts, surgical navigation, and other areas of dentistry[45, 46]. Although the use of 3D reconstruction technology has disadvantages such as the requirement of operator experience [33], varying quality of different scanners [43], and high initial cost [40, 47], the technology has played an important role in dental treatment. Overall, the digital revolution has fundamentally changed the dental industry.

However, to date, no study has sorted out the 3D reconstruction technology widely used in dentistry, summarized the advantages and disadvantages of the techniques, and conducted a comprehensive analysis of the application areas of the techniques. This has led to multiple problems such as confusion in the classification and definition of existing techniques, lack of information sharing between personnel in the field of dentistry and computer vision, duplication of technical research or uncertainty of research directions, and misfit of multiple techniques and multiple application scenarios.

The main focus of this review is to provide a comprehensive understanding of how 3D reconstruction and their subcategories can be used and developed rationally in dental practice by summarizing, analyzing, and reviewing well-developed 3D reconstruction technology in dentistry from various perspectives, including the development, technical principles and characteristics, and application scope of each type of technique.

The role is, but not limited to, enabling clinical professionals and researchers to quickly gain a comprehensive and accurate understanding of 3D reconstruction technology; to provide them with references from the perspectives of technical characteristics and the application and research status of each technique in various fields when selecting the appropriate technique for specific conditions; to inspire technology researchers to find breakthroughs in future research; and to provide ideas for the future development and more application scenarios of dental 3D reconstruction technology.

## Material and methods

### Search strategy

The data for this review were primarily obtained from two academic databases: IEEE Xplore, known for its variety of research results on 3D reconstruction technology, and PubMed, which encompasses a vast amount of literature in all fields of medicine. Google Scholar, a powerful web search engine, was used as a complementary tool. The searched articles have no language restrictions. No publication date restriction was applied and searching were performed until February 2023. Literature search and processing were done independently by two researchers under strict supervision. The search strategy for the databases was as follows:

1. 3D reconstruction technology [Title/Abstract] OR 3D reconstruction technology [MeSH Terms] OR 3D reconstruction techniques [Title/Abstract] OR 3D reconstruction techniques [MeSH Terms] OR 3D imaging technology [Title/Abstract] OR 3D imaging technology [MeSH Terms] OR 3D surface imaging technology [Title/Abstract] OR 3D digital technology [Title/Abstract] OR Surface digitization technology [Title/Abstract]
2. For each sub-category:
  1. Laser Imaging [Title/Abstract] OR Laser reconstruction [Title/Abstract] OR Laser scanner [Title/Abstract] OR Laser 3D Imager [Title/Abstract]
  2. structural light [Title/Abstract] OR structured light [Title/Abstract]
  3. Time-Of-Flight [Title/Abstract] OR TOF [Title/Abstract]
  4. confocal laser scanning microscope [Title/Abstract] OR CLSM [Title/Abstract] OR confocal microscope [Title/Abstract]
  5. optical coherence tomography [Title/Abstract] OR OCT [Title/Abstract]
  6. active wavefront sampling [Title/Abstract] OR active wavefront sampling [Title/Abstract] OR active wave array sampling [Title/Abstract]
  7. passive vision [Title/Abstract] OR monocular cameras [Title/Abstract] OR binocular cameras [Title/Abstract] OR multiocular camera [Title/ Abstract]

### Inclusion and exclusion criteria

Inclusion criteria: scientific papers with peer-reviewed articles published in journals, conferences, and symposia were considered. Our article reviews scientific papers on the 3D reconstruction of human anatomy in the field of dentistry, such as intraoral tissue structures, facial and soft tissues, and cranial bones.

The exclusion criteria were as follows: Research in the direction of materials science, such as cell culture experiments on three-dimensional biological material or research on the application of three-dimensional printing of biological material; research at the microscopic level, such as microscopic imaging of molecules or cells; related to 3D reconstruction technology focused on non-dental medicine areas; articles that do not study 3D reconstruction technology from the perspective of technological development, but only use technology as a means of 3D imaging and do not specify the brand of equipment used or the type of technology used.

### Data collection and extraction process

**Fig. 1:**
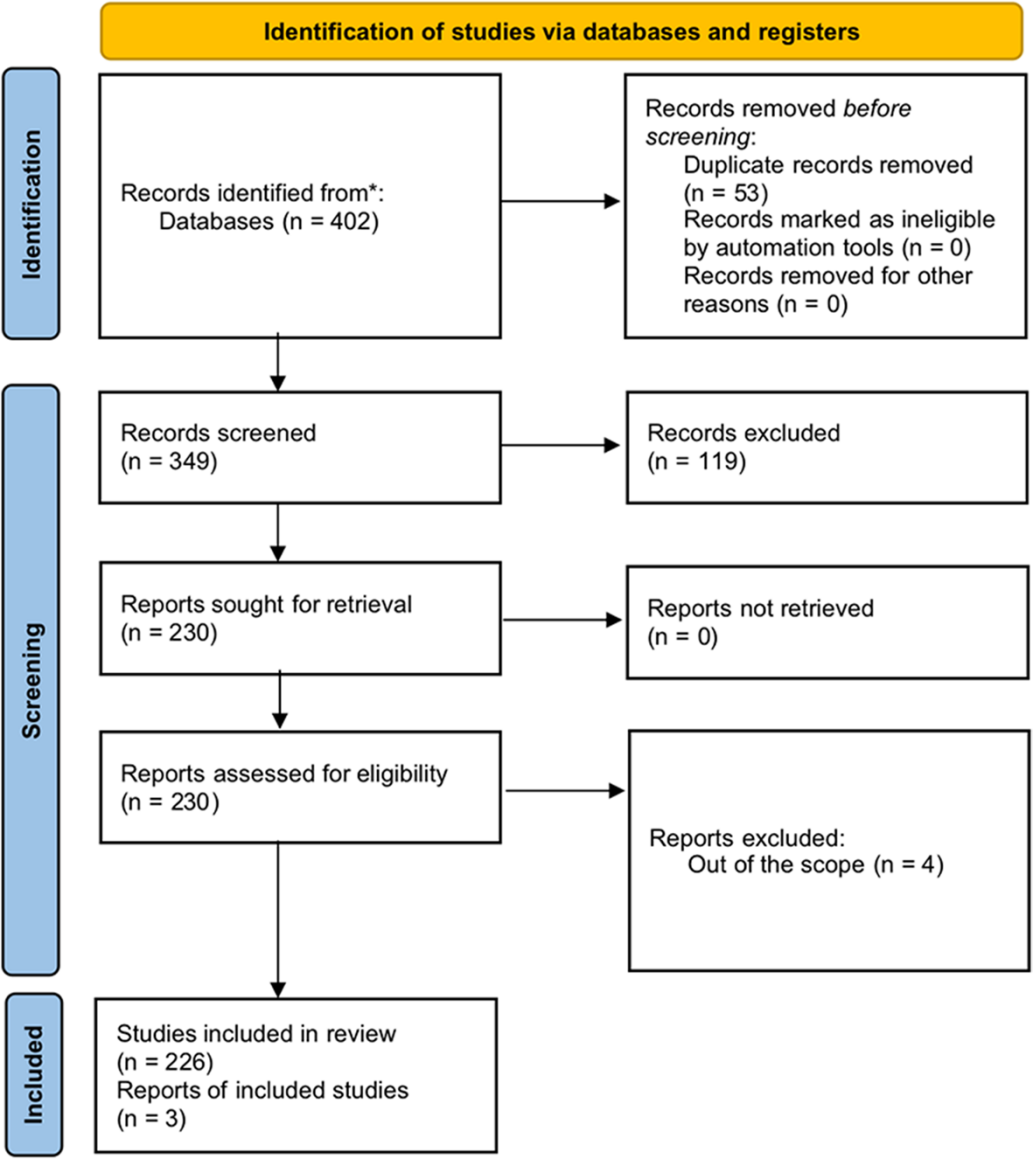
Prisma 2020 flow diagram representing the study selection process

Several electronic searches were screened independently and in a blinded manner by two researchers (YC and XH). If the content of the article can be judged to be relevant to the research objectives based on the information in the title and/or abstract, the full text can be retrieved and read independently by the searcher, provided that the final included article meets the inclusion–exclusion criteria. In addition, the date of publication, basic information about the journal and its field, type, and quality of the study and article are also important criteria for assessing the quality of articles. A flow diagram of the process of identification, inclusion, and exclusion of studies is presented in Fig. 1.

Basic information about the article (type, year of publication, affiliated journal, and its information), author information, study type and design quality, sample number, information about the test subjects (subject characteristics of human-based studies or target types and commercial brands of non-human studies), study design (scanning method used or commercial brand of scanner, times and duration of scans), operation quality (environment, participants, standardization and consistency of the investigator's operation), statistical methods, and the authors’ conclusions and disclosure of conflicts of interest are data that researchers focus on. Two researchers recorded their independently extracted data on a spreadsheet and then compared the results. A third evaluator(WL) will be consulted if there are intractable differences.

### Classification of 3D reconstruction technology and multi-environment applications

**Fig. 2:**
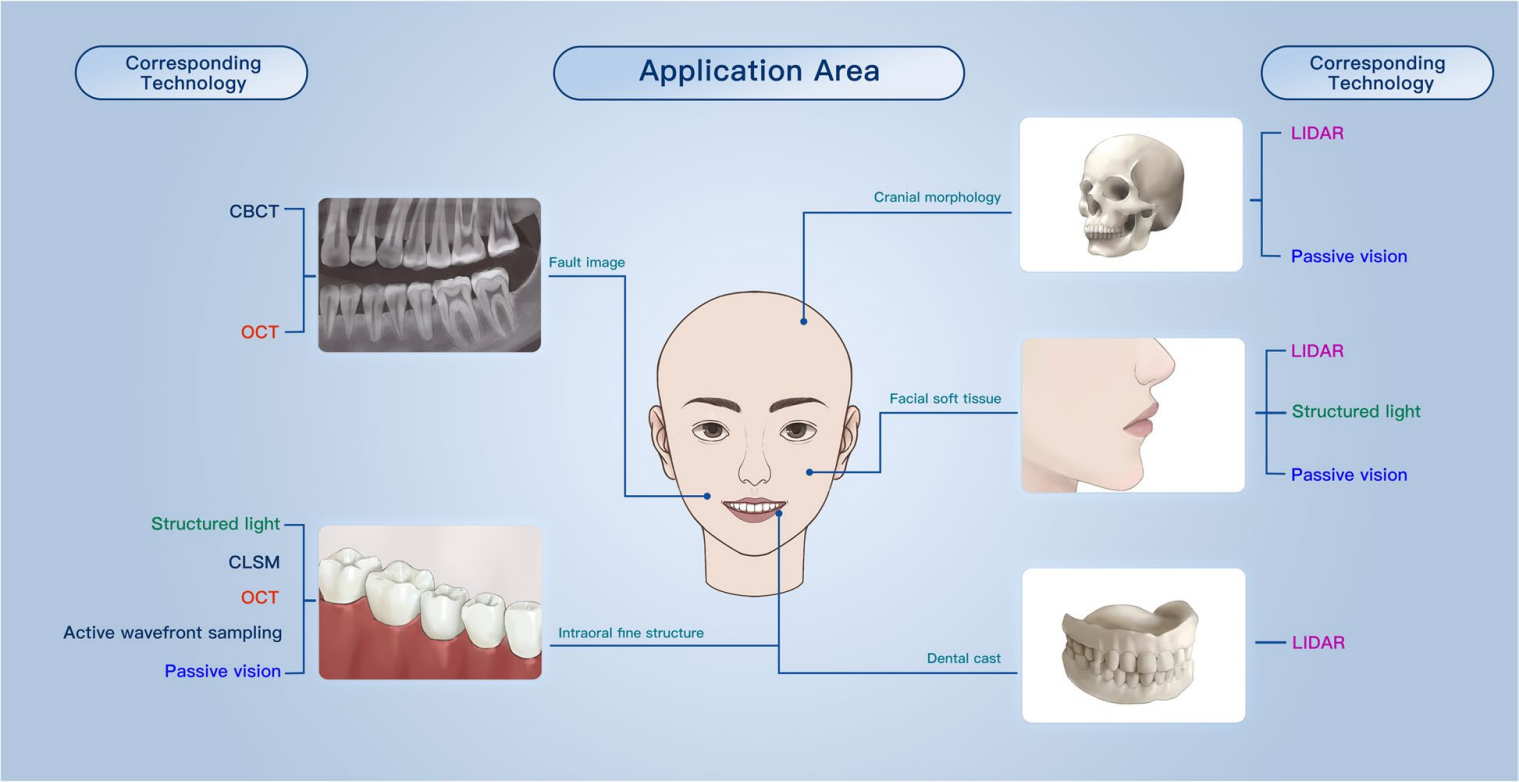
Selection schemes for 3D reconstruction technology in different application areas

Before being applied in the healthcare industry, 3D reconstruction technology has been widely used in other industries [48]. However, in dentistry, the application of this technology still encounters new challenges. The complexity of different fine anatomical regions and the differences in principles or performance advantages and disadvantages of various scanners [49] have led to the adaptation of different 3D reconstruction technology for different specific medical environments [8]. Therefore, since the introduction of [50, 51] 3D reconstruction technology in the 1980s, various technologies have gone through long and complex processes of development and improvement, forming their own characteristics (Fig. 2).

In this review, we have selected the categories of 3D reconstruction technology that are relatively commonly used in dental treatment and, to the best of our knowledge, we have provided a comprehensive, detailed, and accurate classification of these 3D reconstruction technology. The classification results are presented in Table 1. Some researchers have also investigated the possibility of using fusion methods, and such developments have made it possible to produce better 3D reconstructions of objects that are precisely rich in feature details, compensating for the shortcomings of individual imaging methods.

**Table 1:** Classification of 3D reconstruction technology. Classification is based on the principle of the technique and its practical application in dentistry

Data collection equipment types			
Proactive	Reflection-type	Triangulation	Laser Imaging Detection and Ranging (LIDAR)
			Structured light technology
		Time-of-Flight (ToF) method	
		Confocal laser scanning microscope (CLSM)	
		Active wavefront sampling	
	Transmission-type	Cone beam CT (CBCT)	
		Optical coherence tomography (OCT)	
Passive	Monocular camera		
	Binocular camera		
	Multiocular camera		

The classification results are presented in Table 1

In the following paragraphs, this article will introduce each mainstream category separately.

### Laser Imaging Detection and Ranging (LIDAR)

**Fig. 3:**
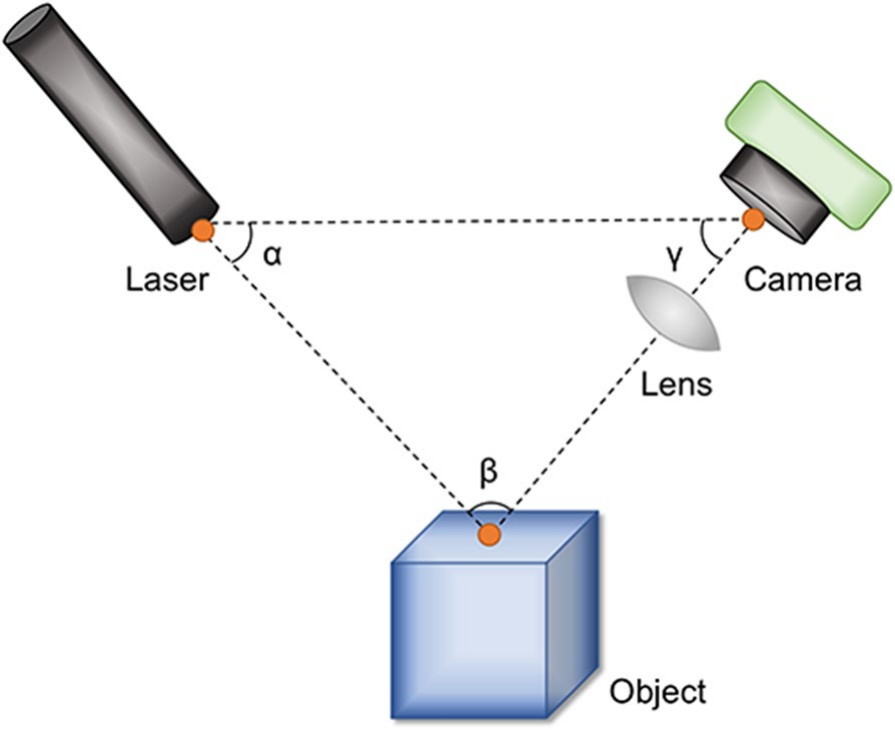
Diagrammatic representation of the LIDAR device. Using a laser light source, target object, and camera, the data can be converted into simple x, y, and z coordinates, and the positions of all the object points can be calculated in a 3D space

**Fig. 4:**
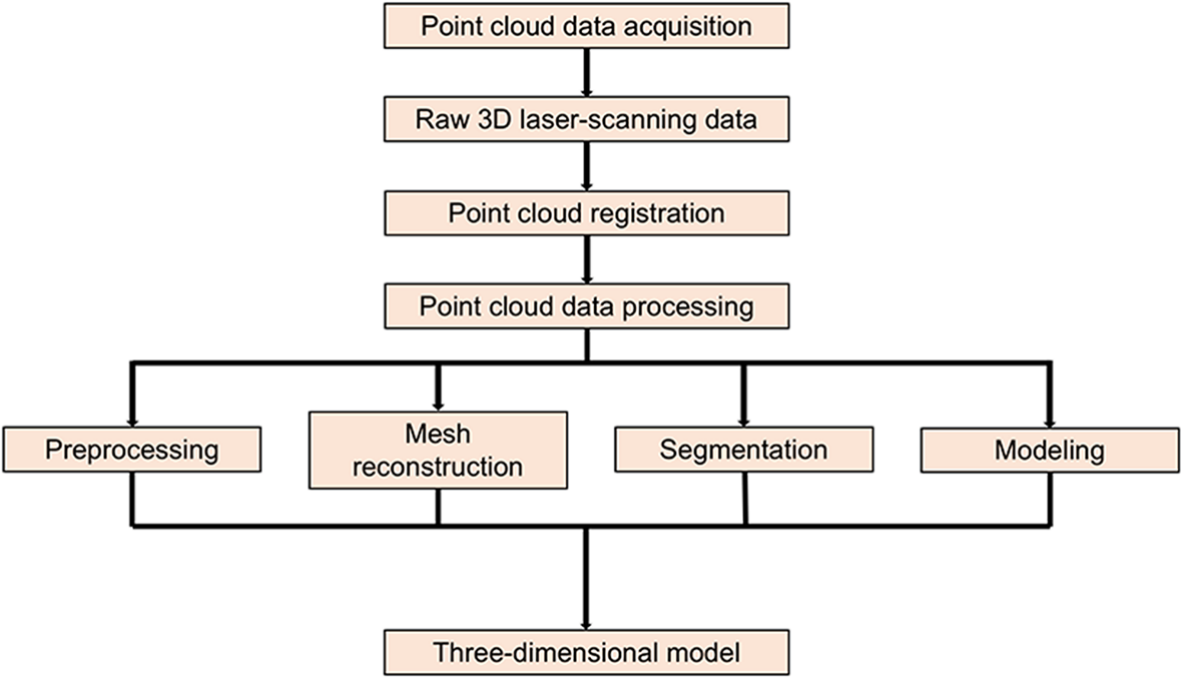
Flow chart of Laser Imaging Detection and Ranging (LIDAR) technique

Laser imaging detection and ranging (LIDAR) uses laser rangefinders to perform real target measurements. Its main role is to measure the exact depth of a single point on a target object and synthesize the data. Laser imaging technology uses optical principles and is essentially an active stereoscopic technique (Fig. 3) [8, 43, 52, 53]. A brief flow chart of the LIDAR is shown in Fig. 4.

It is worth mentioning its principle which dictates that laser scanners have the ability to generate 3D point clouds accurately. This incurs huge costs [9, 16] and a large amount of data prediction [9, 54]. Calibration largely determines the quality of the scanned results, and this step requires well-trained operators [55]. Moreover, the point cloud of the object obtained by the laser scanner is usually not sufficient for application; therefore, it needs to be processed in several ways. The processing of these post-scan data is time-consuming, which is one of the most prominent drawbacks of laser imaging. In addition, measuring recessed parts or parts with complex shapes using laser imaging may cause greater errors [56]. More importantly, after years of research, laser imaging is generally considered clinically effective [33, 57].

The currently known advantages and disadvantages of laser imaging technology in dentistry are listed in Table 2.

**Table 2:** Advantages and disadvantages of 3D reconstruction technology for each category

	Advantages	Disadvantages
Laser Imaging Detection and Ranging (LIDAR)	Quick Capture Speed [52, 53] High accuracy and good repeatability of measurements [16, 58-60] Effective resistance to external disturbances [8, 43] High resolution [43] Good portability [8, 43] Medium photorealistic quality [8, 19]	Algorithms requirements [16] Longer post-scan time [18] Expensive [16] Safety issues with exposure to laser beams [18] Prone to damaged transmitter [16] Loss of laser return [61] and deficiencies in short-range measurements [8, 43, 54]
Structured light technology	High accuracy and good repeatability of measurements [62-83] Strong resistance to external disturbances [16, 65-73], excluding light sources [61] Rapid post-scan processing [16, 70] Convenient [16] High photorealistic [62-64, 79-83] Low energy consumption [16]	Varying resolution quality [73] Algorithms requirements [16] Only for close range targets [73] Prone to damaged transmitter [16]
Time-of-Flight (ToF) method	Quick Capture Speed [16] Simple algorithm [16]	Low measurement accuracy [16] Low resolution [16] High energy consumption [16]
Confocal laser scanning microscope (CLSM)	High measurement accuracy [44, 84-89] High image clarity [44, 84-91] and good expression of details [84-89]	Low capture speed with the use of layer-by-layer scanning [44, 91] Algorithms requirements [91] Long processing time [91] Expensive [89, 91] High energy consumption and complex equipment structure [89, 91]
Optical coherence tomography (OCT)	Very high resolution [92-96]	Tomography results in a low capture speed [92-96] Algorithms requirements [92] Equipment space limitations [94] Imaging depth limitation [97, 98]
3D imaging based on a passive vision	No requirements for scenes [99-101] Convenient [16] Real-time feedback of imaging results [102]	Algorithms requirements [99] Difficult to operate [99]

The currently known advantages and disadvantages of laser imaging technology in dentistry are listed in Table 2

The advantages and disadvantages of this technology, as applied in dentistry, are listed in Table 2

The advantages and disadvantages determined by their technical principles are listed in Table 2

More details on the advantages and disadvantages of their current use in dentistry are listed in Table 2

The current advantages and disadvantages are listed in Table 2

The main advantages and disadvantages of the field and the differences between the three types of passive visual 3D imaging techniques are listed in Tables 2 and 3

### Application development of LIDAR

Laser 3D imaging was the first ever 3D imaging technology to originate, and in the late 1960s [103, 104], it became the backbone of technology for providing accurate 3D models of surfaces, especially in the field of dentistry.

Laser imaging technology pioneered the digitization of the human face[105-108], and its reliability was confirmed many times in subsequent experiments [105, 109-112]. And its technical shortcomings were overcome one by one, leading to practical applications and the establishment of today's position [43, 58-60, 113-115].

### Main application areas of LIDAR in dentistry

#### Registration of cranial morphology

In recording cranial morphology, laser imaging is widely favored by physicians and researchers because of its simplicity, portability, and large amount of data, offering a wide range of possibilities for detailed and accurate analysis of the entire craniofacial complex and virtual treatment [43], which can play an important role in both clinical treatment and complementary research. For example, Xiaojun et al. [116] combined laser scanning with other techniques to develop a computer simulation system for mandibular movements. Terajima et al. [117] combined a non-contact laser scanner to form a new four-dimensional analysis method for oral and jaw function. In the clinical application by Ivanov et al. [118], the laser scanner made an excellent contribution to the imaging of the upper jaw. Jurda et al. [119] also used laser imaging techniques to record cranial morphology.

#### Registration of human face and soft tissue

The inherent advantages of fast capture, resistance to interference, and high resolution, as well as the historical direction of the technology, dictate a high degree of suitability of laser imaging for face and soft tissue imaging.

A large body of literature demonstrates that laser imaging is widely used in this field [120-123], and the accuracy of laser imaging for soft-tissue imaging has been widely noted and repeatedly displayed. Moreover, laser scanners have easy applications and can create 3D images with extraordinary applications.

3D capture of the human face and soft tissue morphology is an indispensable support for treatment planning and performance of plastic and reconstructive surgery, especially cleft lip and palate (CLP). While most studies on soft tissues are performed by laser surface scanning, as expressed by Canto G. [33], the number of publications listed on PubMed on 3D imaging of CLP patients is steadily increasing. The main relevant scanners currently in the market are FastSCAN™ [8] and Solutionix [19]. It is worth noting that although LIDAR uses non-ionizing radiation for imaging, laser damage to the eyes should be considered when using it for 3D reconstruction of human face [108]. Dose limitation and optimization of protective measures should be further investigated.

#### Registration of dental casts

Data obtained from dental models are useful for the diagnosis and determination of treatment plans [43, 124]. Researchers have also used laser imaging to obtain the 3D morphology of dental models, such as by Yousef et al. [125-128]. Noh et al. [129] noted that the accuracy of integrating dental images from laser scans into maxillofacial Cone beam CT(CBCT) images could be improved [130]. The Minolta intraoral scanner developer [124, 131] is the most typical example, and the accuracy and scanning speed has been increasing in a short period [124, 132-134]. In conclusion, in the current field of intraoral scanning, LIDAR integrates the advantages of portability, speed, ease of operation, simplicity, and small size, and has also become a common technology.

#### Structured light technology

**Fig. 5:**
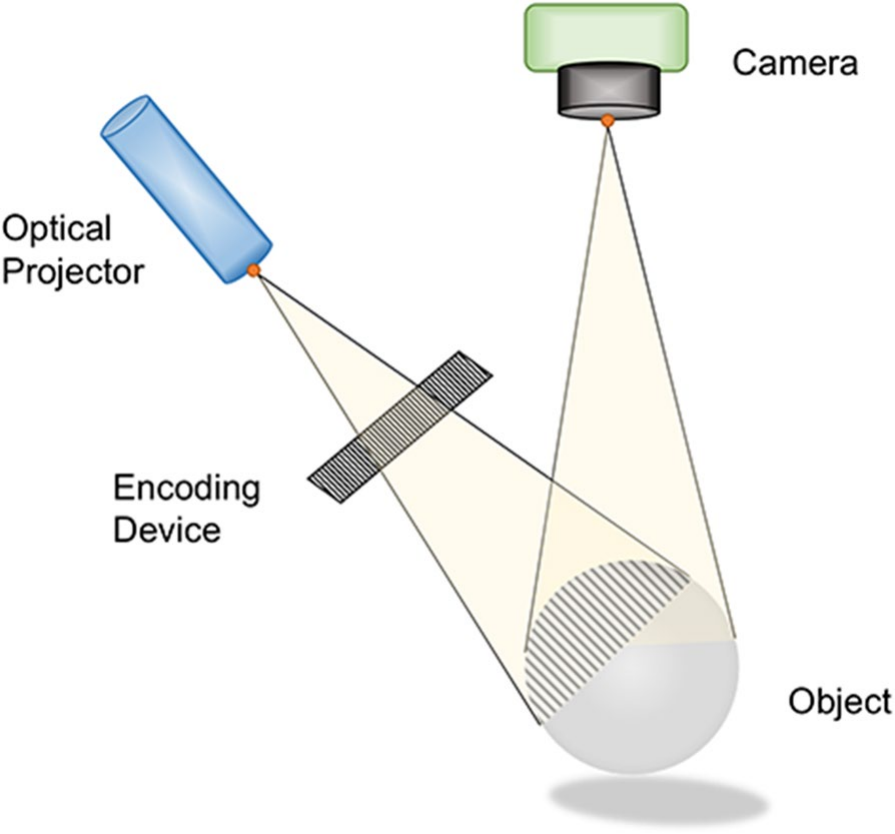
Diagrammatic representation of the structured light device. The projector emits a "structured" light pattern that distorts and deforms when light hits the surface, generating feature points

Structured light technology is another widely used class of 3D imaging technology based on the principle of optical triangulation, and consists of optical projectors, cameras, and computer systems to form a structured light 3D vision system (Fig. 5) [16, 53]. It emits light with characteristic points to an object with a smooth and featureless surface and extracts the depth information of the object based on the stereo information in the light. Different studies have evaluated the accuracy of various scanners based on structured light technology, all with better results [62-64].

It is considered one of the mainstream types of 3D reconstruction technology mainly because it can capture the shape of the target realistically and accurately and create a simulated image with a good balance of other properties. And in active vision-based 3D reconstruction technology, it has relatively strong resistance to environmental interference but is still affected by ambient light [16, 65-73]. The advantages and disadvantages of this technology, as applied in dentistry, are listed in Table 2.

#### Application development of structured light technology

The development of structured light technology occurred slightly after LIDAR, originating around the 1970s [74-78], but from the beginning, it has received a lot of attention from researchers around the world. Various breakthroughs in structured light technology have been described in detail [16], and this study focuses on the development of this technology in the field of dentistry.

As with laser imaging, early developments have focused on facial imaging [66] and made a number of significant breakthroughs [65-72, 79-83]. In recent years, Piedra-Cascón et al. [135] verified that specific dental optical scanners based on structured light scanning technology have significantly higher realism and accuracy than non-dental structured light scanners, affirming the process of taming the technology by researchers in the field of dentistry.

The analysis of its research potential will be described separately in later sections according to the classification of technology application scenarios.

### Main application areas of structured light technology in dentistry

#### Reconstruction of human face and soft tissue

As mentioned earlier, the characteristics of the technology and the directed research have led to the excellence of structured light technology for 3D imaging of the face. This conclusion has been repeatedly confirmed in studies [136, 137].

It was the first technique proposed for the complete capture of soft facial tissue, facial skeleton, and dentition (maxillofacial triad) and can be integrated on the basis of imaging results to construct maxillofacial models that can be used for surgical design and efficacy analysis in orthodontic treatment [138]. Subsequent studies have progressively complemented its powerful ability to measure soft tissue thickness [139] and accurately assess complex areas or dynamic soft tissue profiles [140, 141]. In the field of scientific research, the step involving the extraction of 3D data of the face, many researchers [142-153] choose to use structured light technology or scanners based on this technology, reflecting its wide acceptance in dentistry. 3dMD system is one of the classic facial imaging devices used in dentistry. The main imaging principle is structured light combined with active visual 3D imaging techniques, which is discussed later, and this suggests that combining two mainstream technologies for face imaging may be a mutually improving approach.

#### Fine structure of the oral cavity

Structured light technology cleverly solves the optically challenging defect of a smooth and featureless object surface that makes it difficult to image and is especially suitable for targets with flat surfaces, single textures, and insignificant color variations, such as the smooth face of teeth. Moreover, the technological principle is simple and easy to implement. As mentioned earlier, most relevant research in the field of dentistry has focused on improving the accuracy of structured light technology, which can now produce precise and critical anatomical information and participate in various minimally invasive clinical procedures [154]. Therefore, structured light technology can meet the requirements of microscopic 3D morphology for high-precision measurement, and has a certain potential for 3D reconstruction of fine structures inside the oral cavity.

The aforementioned advantages dictate its use in orthodontics, prosthetics, and other disciplines that require the acquisition of fine morphology of intraoral tissues. Nagata et al. [21] showed that an intraoral scanner with structured light technology as the main principle could be used for imaging up to three units of implant prostheses, although the sample number of the study was its obvious limitation. Based on this study, they also suggested that applying this technique to the development of intraoral scanning has the potential to reduce the risk of medically-derived infections, but further research is needed.

In summary, considering the combination of structured light technology with other techniques to improve the imaging results on smooth intraoral planes is also one of the directions worth investigating. For example, the combined use of structured light technology and confocal microscopic imaging is currently the mainstream technology for intraoral scanners, which is also described in later sections.

#### Insufficient funding

The high price has been one of the non-negligible drawbacks that limit the use of scanners in clinical trials in private hospitals and private hospital dissemination. As proposed by Nowak et al. [155], the development of scanners should focus mainly on reducing time and cost. Currently, structured light technology is frequently chosen and applied in the research of developing low-cost instruments [156-158]. This hints that it may be the preferred technology in case of insufficient funding, and it may become the mainstream technology for developing low-cost scanners in the future.

#### Time-of-Flight (ToF) method

The Time-of-Flight (ToF) method is an active triangulation technique. Pulsed light is emitted from the transmitter to the object, reflected by the object, and stopped when the receiver receives the reflected light. The computer determines the distance of the object based on the time difference between the transmission and reception and determines the resulting depth information. Then, the data of each detected point is assembled to form huge 3D point cloud information to reflect the target's morphology. The advantages and disadvantages determined by their technical principles are listed in Table 2.

The characteristics of the technique, such as spatial limitations and low accuracy, determine its unsuitability as a primary technique in dentistry. It is more suitable for the 3D imaging of large objects. However, it can be used as an auxiliary technique to enhance the performance of other 3D reconstruction technology, which suggests a possible direction for its development. The Kinect 2.0 sensor, introduced in 2014, is a prime example of the combination of technologies as described above, in which ToF plays a role in enhancing the ability to acquire depth information on the surface of the object.

#### Confocal laser scanning microscope (CLSM)

**Fig. 6:**
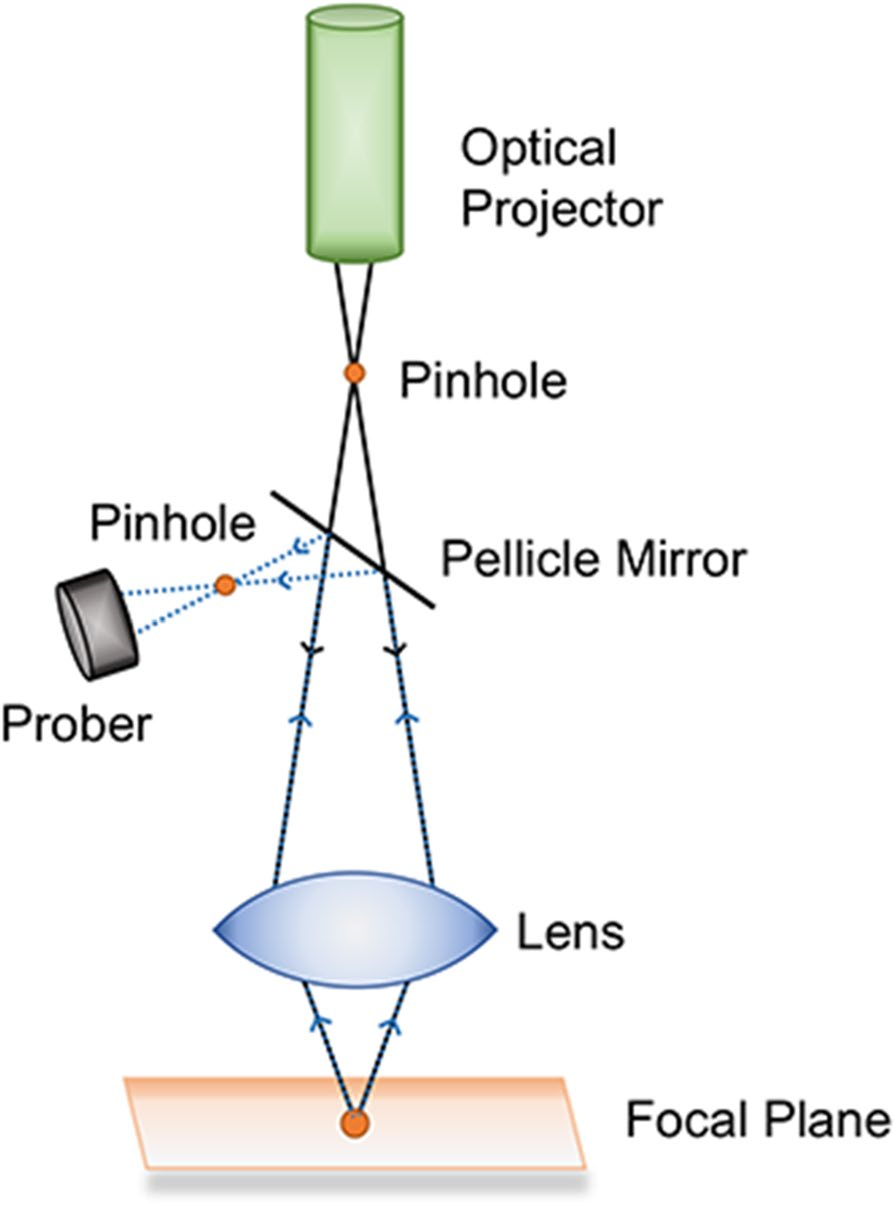
Diagrammatic representation of the CLSM device. There are often various lenses involved in changing the optical path. Only the morphological data of the teeth on the focal plane are obtained

Confocal microscopic imaging is a technique used to acquire focused images from a selected depth. Its core devices include an illumination pinhole placed behind the light source and a probe pinhole placed in front of the detector. In the imaging process, the direction of the optical path is changed mainly by adjusting the equipment, and a layer-by-layer scanning mode is used to obtain images of different layers at different points on the target surface, so it is also referred to as “optical slicing”. Finally, the 3D morphology of the scanned target is computationally reconstructed (Fig. 6).

The most significant advantage of the technology itself is the superior imaging performance [90]. Besides, with this technique, the surface profiles of opaque specimens can be reconstructed, and the internal imaging of non-opaque specimens can be obtained. In addition, the images can be viewed continuously on the screen during the scanning process. The user can visualize the completion of the scan to ensure that no area is missed [90, 91]. However, due to the use of layer-by-layer scanning technology, the speed of 3D reconstruction (including the speed of information acquisition during scanning and the speed of data post-processing) and the high requirement for algorithms also lead to obvious shortcomings. More details on the advantages and disadvantages of their current use in dentistry are listed in Table 2.

#### Application development of CLSM

Despite the late origin of confocal microscopy [91] and the lack of extensive technical research, the technology itself is particularly favored in the field of three-dimensional reconstruction of dentition owing to its significant advantages of microscopic properties and extremely high definition and accuracy in obtaining images. As mentioned earlier, most orofacial scanners on the market today use a three-dimensional imaging principle that combines CLSM and structured light technology. CLSM has become one of the dominant technique for intraoral imaging.

For example, Sirona was one of the first scanner developers to apply CLSM, playing an important pioneering role by combining the principles of optical triangulation with technical innovation, dynamic imaging optimization, scan speed improvement, and real-time enhancement of CLSM. iTero, which entered the market later, is one of the most widely used intraoral scanners in clinical use today, capturing image information with greatly improved clarity and scanning speed [44, 84, 85].

From the point of view of the technology itself, the different projection images and the corresponding data processing procedures are important factors that widely affect its application performance and are the focus of current and future research [84, 85, 91].

### Main application areas of CLSM in dentistry

#### Dental 3D reconstruction

As the main technological pillar of intraoral scanners (IOS), CLSM, which excels in accuracy and detail portrayal, is widely used in clinical and scientific research in dentistry for various intraoral tissue 3D reconstructions, represented by applications in orthodontics and prosthodontics.

In a retrospective study of a large number of studies evaluating intraoral scanners, Jabri et al. [57] indicated that the use of CLSM technology maximizes the accuracy of intraoral scans and is an ideal tool for converting dental plaster models to digital models. This allows it to be currently used in orthodontic treatment such as the precise analysis of the patient's dental morphology and the development of treatment plans. It can also be used for almost all treatment sessions and types of restorations such as resin inlays and fixed partial dentures [87] in prosthodontic treatment. And the scientific literature confirms that optical impressions are clinically similar to conventional impressions in terms of accuracy and fidelity, which continue to improve [88, 89]. In addition, in a cross-sectional comparison study of multiple techniques [24], the scanner with CLSM as the core technology showed better imaging results in the transitional portion of partially missing to completely missing teeth on the model. This suggests that it may have more application scenarios than other intraoral scanning techniques, such as making precise intraoral scanning of the full arch possible.

In addition, CLSM is favored by researchers for obtaining color 3D models [159]. The color impression of intraoral tissues has also been an important property of the newly developed intraoral scanner in recent years.

#### Dental microwear and micro lesions

Due to the excellent contribution of CLSM in microscopy and its ability to represent great details, DeSantis et al. [160] in 2013 confirmed the importance of the former in monitoring dental microwear by comparing the accuracy of confocal microimaging techniques with 2D imaging techniques in reproducing dental microwear images in herbivores. One year later, Maia et al. [161] confirmed that CLSM could be used to reproduce a 3D model of the eroded surface of tooth enamel with good results.

Since then, CLSM has been considered an effective tool for imaging dental microwear and micro lesions. For example, Austin et al. [162] used it for the study of enamel erosion and remineralization properties, and Hara et al. [163] used CLSM to test enamel 3D surface texture outcomes to study the pathological alterations of erosive tooth wear. With the aid of CLSM, Mullan et al. [164] also proposed a method to accurately measure the surface texture of human natural teeth.

#### Active wavefront sampling

**Fig. 7:**
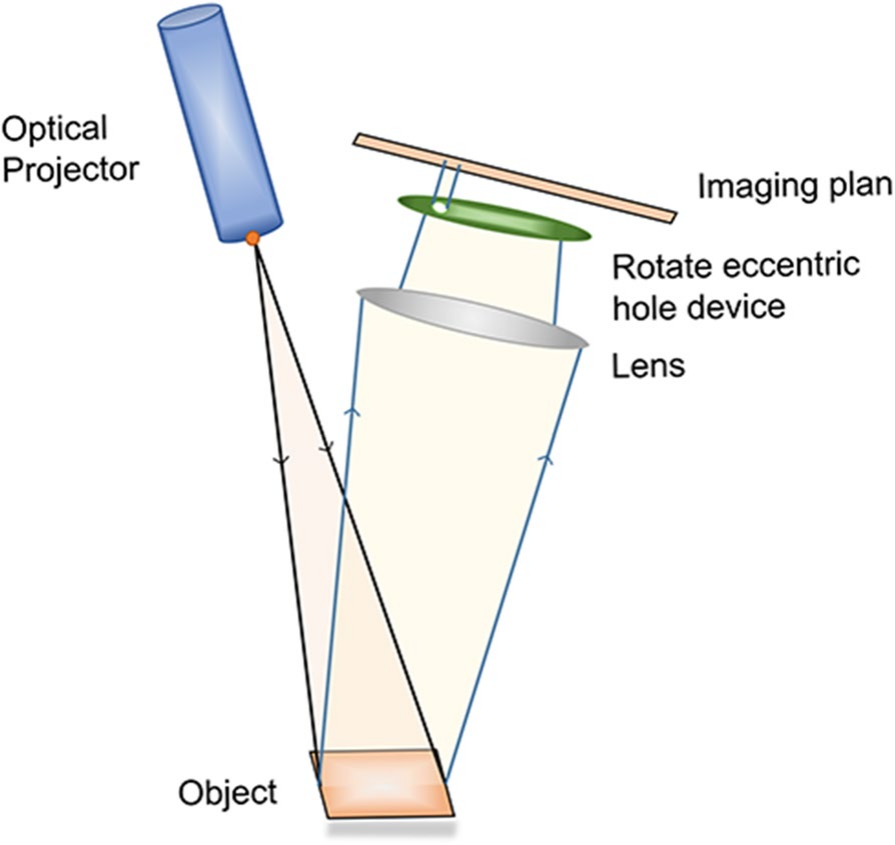
Diagrammatic representation of an active wavefront sampling. The calculation of the focus and defocus depth is based on the measurement of the main optical system

The active wavefront sampling device consists of an emitter, lens imaging system, and sensor. The beam emitter projects light onto the scanned object, and the image reflected by the target passes through the lens imaging system and is selectively filtered using a rotating eccentric-aperture device. The light filtering effect of this device can well prevent the images of different areas of the tooth surface from overlapping, thus improving the spatial resolution of the images. The reflected light is projected onto the sensor to form an out-of-focus image of a circular trajectory, and the spatial position of each measurement point is calculated in combination with the known information of the optical path to obtain the 3D information of the scanned target (Fig. 7) [165, 166].

The large amount of data and the high speed of information acquisition are the most significant advantages of active wavefront sampling. Twenty 3D datasets can be captured per second,embodying over 10,000 data points in each scan [167]. However, it does not show significant superiority in this respect compared to other technologies [38]. The most prominent drawback is that when using a scanner with active wavefront sampling as the core technology, the particular powder needs to be sprayed on the tooth surface to form a homogeneous layer after the mouth rinsing and air drying. This requirement makes the use of active wavefront sampling significantly less convenient and less comfortable for the patient, and the powder itself can affect the accuracy of imaging. Moreover, it has high equipment requirements and is difficult to make major breakthroughs or correct shortcomings at the technical level due to the limitations of its principles.

Combined with the above factors, this technique is relatively unpopular in dentistry-related research. Nevertheless, there are still mainstream intraoral scanners that primarily choose to use this type of technology, represented by the Lava COS and True Definition Scanner [38, 167, 168]. According to studies on the application of these scanners, active wavefront sampling have shown weak competition for applications in recent years and have been used only for intraoral scanning.

Overall, the applications and research related to dentistry, with active wavefront sampling as the main technology, are at a disadvantage and have not shown a significant trend of becoming popular. The research significance and development prospects are relatively pessimistic. But it can be considered to develop new technologies and application models that combine with other technologies in some application scenarios that need to improve imaging accuracy and data content.

#### Cone beam CT (CBCT)

**Fig. 8:**
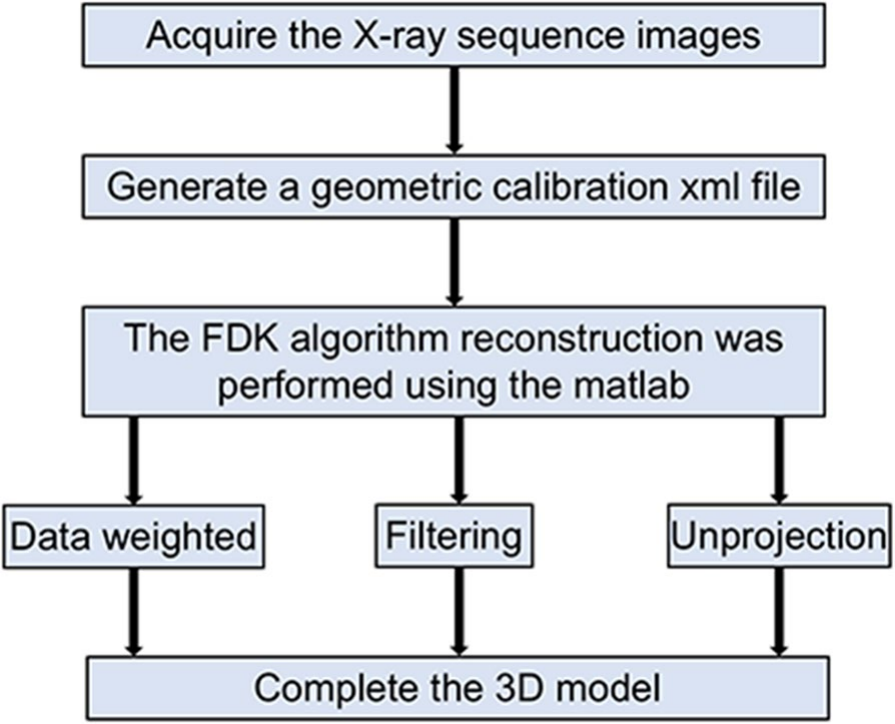
Flow chart of the CBCT 3D reconstruction

CBCT acquires serial X-ray images through rotational scanning and generates 3D images using a 3D reconstruction algorithm. The 3D reconstruction process is illustrated in Fig. 8.

The vast majority of diagnostic and therapeutic applications in the field of dentistry require the use of this technology, which is primarily used for pathological examination of tooth roots, such as periapical [169] and root resorption [170], pathological imaging of the dental pulp, such as root canal therapy, imaging of bones in cranial segments, such as the mandible [92, 93], and soft tissue evaluation, such as the gingiva [94]. It is worth noting that radiation exposure is its most significant feature compared to other techniques. The radiation protection principles of justification and optimisation are emphasized by International Commission on Radiological Protection. However, there are significant differences in radiation dose levels between even the same equipment model, which leads to instability in protection [95]. Reducing the harmful effects of ionizing radiation by means such as optimisation of imaging parameters and equipment is one of the priorities for future research in CBCT.

### Optical coherence tomography

Optical coherence chromatography (OCT) is a non-invasive, high-resolution optical imaging technique that generates cross-sectional images of objects in real-time based on the interference between the signal from the object under study and a local reference signal [96]. OCT has a very high resolution that is almost hundred times higher than that of confocal microscopy [96, 171]. Therefore, OCT is a new diagnostic imaging technique with many potential dental applications [172].

According to the published literature, the main development period of OCT application in dentistry started five years ago, but it has already shown a considerable 3D imaging effect and has a desirable developmental prospect. In recent years, there have also been developers of mouth scanners that use OCT technology as the main 3D imaging principle, or add OCT as a secondary technology. These mouth scanners have performed well in several tests [173, 174]. The current advantages and disadvantages are listed in Table 2.

### Main application areas of OCT in dentistry

#### Scanning of dental hard tissues and restorations

Due to its non-invasive, cellular level resolution and tomographic properties, OCT technology has excellent prospects for application in the field of 3D imaging of dental hard tissues and restorations [175].

The earliest use cases [176] where OCT was introduced into dentistry were when Otis et al. [177] and de Melo et al. [178] used it to image tooth composition such as enamel, dentin, and restorations. Subsequent experiments have repeatedly confirmed that OCT can be used for 3D structural imaging and functional diagnosis of human teeth [179], due to the ability to generate high-resolution images of microdamage [180-182].

Although OCT is an emerging technique, it has been widely used. The most widespread application is the use of OCT to detect secondary caries, such as Schneider et al. [183-186] There are also many studies using OCT to observe the tomographic morphology of restorations [185, 187], such as internal gaps [188], in order to evaluate the various properties of restorative materials or to use the 3D images acquired by OCT as a basis for related studies such as material curing scheme selection [189]. Furthermore, the imaging of the dental tissue itself is also one of its extremely valuable application scenarios. Imai et al. [190] used the capability of OCT to clearly image enamel crack and whole-thickness enamel crack to confirm the extension of enamel cracks beyond the dentinoenamel junction. What’s more, it can be used to propose new guidelines for dental-wear assessment [179].

Overall, the frequency of choosing OCT for imaging dental hard tissues and restorations has shown a significant upward trend in recent years, showing optimistic research implications and development prospects. However, different restoration materials result in different degrees of signal attenuation, which causes differences in imaging results [176]. This has been the focus of recent studies related to the application of OCT for restorative imaging [191].

#### Replace or improve X-ray radiography

Notably, one of the most remarkable features of OCT is the ability to produce cross-sectional images of tissue structures. According to Shimada et al. [185] proposed that OCT has higher sensitivity and earlier image changes than dental X-rays in caries detection, which means that OCT is a reliable and accurate method and a safe alternative to radiography.

Erdelyi et al. [97] clearly suggested through high quality radiological studies and analysis of experimental data that OCT is more suitable than plain x-rays for assessing dental problems and applying them in the treatment process, except for bone-related investigations and periodontitis. In addition, OCT is free of ionizing radiation and can achieve imaging levels comparable to destructive high-resolution microscopy. Therefore, it has been shown to provide qualitative images in situations where X-rays are not appropriate, such as in patients with developmental disorders [192]. These studies demonstrate the potential of OCT to replace X-rays in the future.

As a proven depth-resolution imaging technique, many research teams have also explored the feasibility of using OCT to improve X-ray techniques. Erdelyi et al. [193] claimed that the combined use of X-ray and OCT techniques has no drawbacks and can significantly improve resolution. This is one of the directions worth studying in the future.

#### Periodontal tissue and oral environment monitoring

OCT has promising applications in the diagnosis and monitoring of soft-tissue conditions in the oral cavity. This includes an assessment of mucosal [194, 195] status, plaque, and gingival aspects [196]. As early as 2009, Baek et al. [98] used OCT to measure changes in the periodontal ligament and confirmed its accuracy. Subsequent studies have also demonstrated that OCT can produce high-resolution images of periodontal structures [61], including tissue contour, dental calculus, and connective tissue attachment, which can be used for periodontal tissue and oral environment monitoring and early detection of active periodontal etiology.

In addition, researchers have claimed that improvements in imaging depth [61, 192] and the development of intraoral sensors are likely to make OCT a useful technique for periodontal applications, suggesting a direction in which future OCT research should focus.

In conclusion, OCT, as a newly emerged 3D reconstruction technique, has demonstrated good potential but remains to be explored.

#### 3D imaging based on the passive vision

Compared with the other principles of three-dimensional imaging technology introduced previously, this technology is more widely used, the development of which is exceptionally rapid despite its relatively late adoption [99].

Passive visual 3D imaging technology imitates humans using cameras instead of eyes to perceive 3D structures. It involves the acquisition of image sequences by visual sensors (one or more cameras), which generally means acquiring two or more images from different viewpoints. Based on this, the difference between the two viewpoints is calculated using triangulation to perceive the depth information and reconstruct the 3D structure or depth information of the target object. Useful information from the acquired images is extracted, reverse engineering modeling is performed, and a 3D model is created. A noninvasive, non-contact technique with no radiation exposure is used [102].

Since passive visual 3D imaging technology have the significant and important advantage of being virtually immune to environmental interference compared to the active vision technology mentioned above, much effort has been devoted to the research of this technique in recent years. In addition, it has the advantages of lower price and higher real-time performance, and the need for algorithms and the difficulty of operation are probably their overall weaknesses. But according to the number of cameras, passive visual 3D imaging techniques can be classified into monocular vision [197-202], binocular vision, and multiocular vision. The different classifications have some differences in performance in terms of environmental restrictions, reconstruction results, ease of operation, cost and reconstruction speed. In general, with an increase in the number of cameras, the imaging effect is gradually optimized and the application scenarios is wider, but the cost increases significantly, and it is increasingly difficult to operate. Therefore, in developing this technology, the key to research is an improvement based on a good balance between all aspects of its performance [16, 99].

**Fig. 9:**
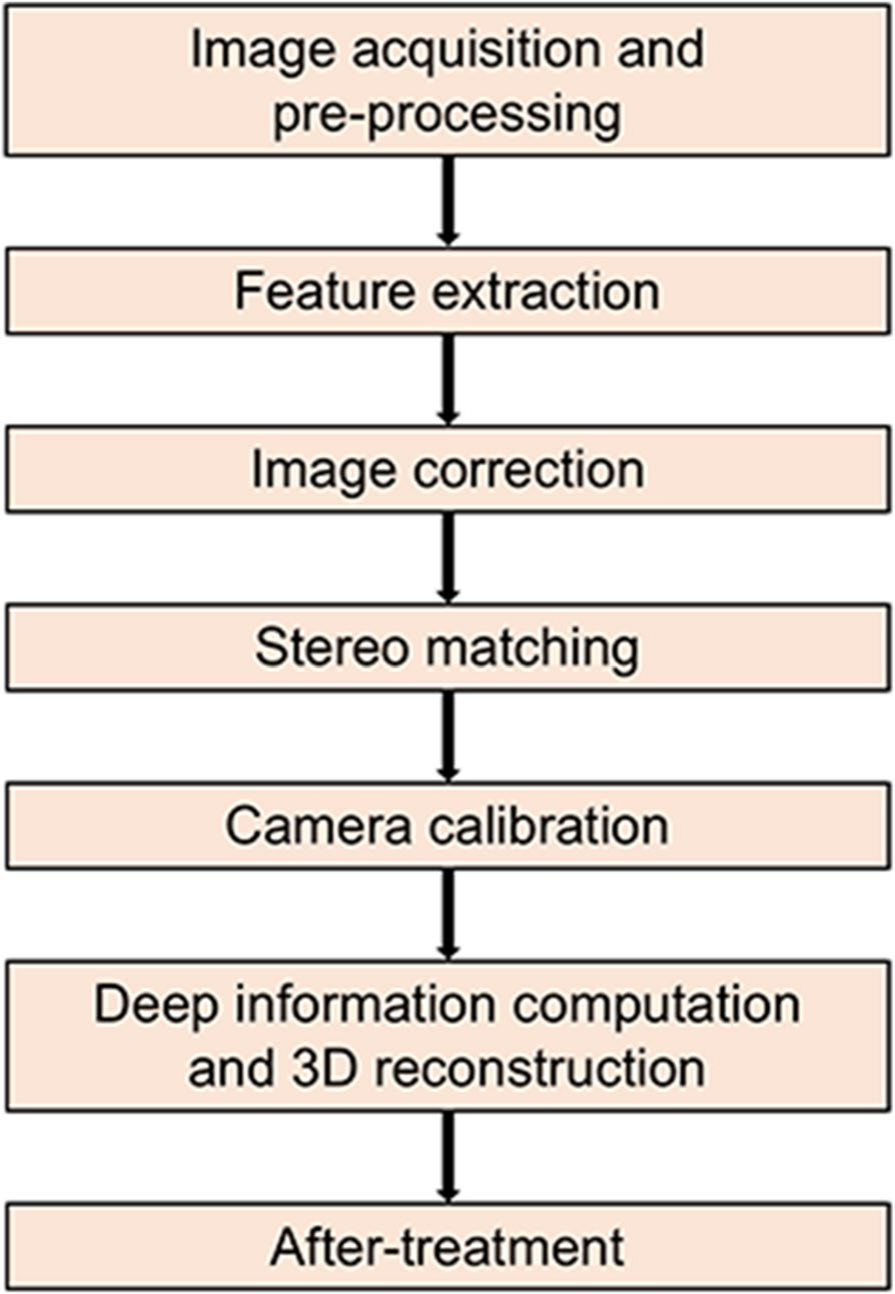
Flow chart of 3D reconstruction based on passive vision

The main advantages and disadvantages of the field and the differences between the three types of passive visual 3D imaging techniques are listed in Tables 2 and 3. The general flowchart is shown in Fig. 9.

**Table 3:** Characteristic comparison of the three passive visual 3D reconstruction technology

Classification	Applied range	Reconstruction results	Ease of operation	Cost	Reconstruction speed
Monocular vision	Wide range of applications	Poor accuracy of depth information	Better control	Cheap price	Short processing time
Binocular vision	Wider range of applications	Consistent and good results	Bad control	High cost	Large volume of operations
Multiocular vision	Suitable for all kinds of scenes with a large field of view	Better reconstruction, more accurate, high recognition accuracy	More difficult to manipulate and control	More expensive	Large volume of operations and long reconstruction time

The main advantages and disadvantages of the field and the differences between the three types of passive visual 3D imaging techniques are listed in Tables 2 and 3

#### Monocular vision imaging technology

Monocular vision is a 3D reconstruction method that uses only one camera [197-199]. Over the years, researchers [200-204] have conducted research on its dynamic imaging, imaging quality, and more. Simple calibration and compact computational efficiency are significant advantages. However, their lack of depth information, the large number of blind areas in the extraction of object appearance data, and the size limitation of the imaging area are shortcomings that still exist [202, 203]. In general, a single camera cannot obtain information for multi-angle observation of the target, and in the case of the 3D imaging needs of dentistry, both extraoral and intraoral tissues have complex morphological structures; therefore, the performance drawbacks described earlier due to the principle of the technology dictate that the technology is currently hardly used in dentistry. Future research on this technology should focus on algorithm improvements and its application in combination with other technologies [205, 206].

#### Binocular vision imaging technology

Binocular vision works using two cameras to mimic human eyes, capturing images of different perspectives of the same target, and then using the principle of triangulation to reconstruct the surface of the target in 3D [207]. The advantage of this system is the ability to obtain a larger range of views and to perform more accurate 3D reconstructions under conditions that match the calculations of more complex algorithms [207]. Because of the balance and compliance of each performance, this class of techniques is the focal part of 3D reconstruction technology with great potential [16] and has been fully developed in favor of its application in dentistry [208, 209]. Currently, 3dMD system, a 3D imager that has an important role in the field of facial imaging in dentistry, uses binocular vision-based 3D reconstruction technology as its main principle.

However, it still has the significant shortcoming that the triangular relationship between the two cameras and the imaging target is difficult to adjust precisely, which leads to practical difficulties [210, 211]. If the angle between the two camera views and the target is too large, more false matches are generated, which requires more complex algorithms. And if the parallax is too small, it reduces the imaging range and the quality of data extraction. This is the focus of the current breakthrough needed for this technology.

#### Multiocular vision imaging technology

Its advantages in terms of application scenario limitations and imaging results are apparent, but due to its late development, the technology has not yet been adapted to the requirements of dentistry. In recent years, the research of this technology in terms of device hardware has gradually become a hot topic [212], but it is still in its initial stage.

### Main application areas of passive visual 3D imaging in dentistry

#### Facial imaging and soft tissue assessment

Passive visual 3D imaging is widely used in dentistry in combination with various other techniques and has a wide range of applications; however, the classic use is facial imaging and soft tissue assessment [213, 214], especially for binocular vision imaging technology. The most prevalent is the 3dMD system [27, 100, 210, 211, 215], which combines passive and active stereo-photogrammetry [100], where the underlying motion of the facial expression and respiration can be ignored because of the very short photo capture time of 1–2 ms [216]. For example, Lau et al. [217] used 3dMD to assess the degree of facial swelling in patients after third molar extraction and Kaba et al. [218] used data generated by 3dMD to assess the effectiveness of medications in reducing swelling. And studies have shown that its precision and accuracy are the highest of almost all facial scanners [210]. In addition, it has great potential for extended use. For example, Xin et al. [216] proposed the use of CT technology in combination with 3dMD, with ease of use and high reliability. From the perspective of research trends, researchers who focus on techniques for acquiring facial features also prefer such techniques as their main research direction [219].

The superiority of passive stereoscopic imaging for facial imaging applications has made it the first choice for orthognathic surgery [220] or the treatment of various other craniofacial anomalies [211, 221]. This technology allows the surgeon to quickly obtain and save the soft tissue morphology of the patient's face at any time of treatment, and to easily perform precise linear measurements and angular calculations of any marker point on the resulting 3D image of the face [222]. On this basis, in the wide range of applications it has received [101, 210, 222-224], the patient's condition can be accurately analyzed and diagnosed before surgery and the surgical plan can be improved, and the soft tissue changes after orthognathic surgery can be accurately and efficiently evaluated and the efficacy demonstrated, providing key reference information for determining the success or failure of treatment.

#### Intraoral scan

Although the suitability of choosing 3D imaging based on the passive vision for intraoral imaging is not as good as the other techniques described above for the same purpose, it has also been shown to be used for intraoral scanning when, for example, only such equipment is available or when a particular situation is considered that leads to severe light interference in the application scenario.

In a study by Tohme et al. [225], such techniques reported the highest accuracy in terms of fidelity and precision of intraoral scanned bodies for all evaluation techniques. Furthermore, this literature mentions the need for future studies to evaluate different types of photogrammetric systems and implant angles, connections, and volumes. However, in the study by Ortensi et al. [226] it was found that the height and width data of the teeth were accurate in the scans obtained by the 3dmd system, but the mesial-distal dimension imaging results of the teeth were prone to distortion.

In conclusion, passive visual 3D imaging techniques have some utility and potential for intraoral implant scanning, but is not a preferred option.

## Conclusion

In conclusion, 3D reconstruction technology have made an undeniable contribution to the digitization of the surface morphology of human tissues involved in dentistry. However, to date, none of these technologies apply to all areas of dentistry. Researchers and physicians need to select the most appropriate instruments corresponding to the 3D reconstruction technology according to the research or clinical situation involved to maximize the convenience and accuracy of 3D imaging. Manufacturers and researchers must target a certain technology category as the main technology pillar for future development and upgrades of the products in specific application areas. Finally, from the point of view of technological development and existing research, the integration of different technologies is the most promising way to overcome their respective limitations, which can lead to more application scenarios and better performance of 3D reconstruction technology.

In addition, the reference to English literature only is one of the limitations of this review. Studies in many other languages, such as Indonesian, are also authentic and reliable, so the deficiencies in references selection may result in information bias [227].

## Data Availability

The datasets used and/or analysed during the current study are available from the corresponding author on reasonable request.

## Abbreviations

3D: Three-dimension
2D: Two-dimension
LIDAR: Laser imaging detection and ranging
CLP: Cleft lip and palate
CBCT: Cone beam CT
ToF: Time-of-Flight
CLSM: Confocal laser scanning microscope
IOS: Intraoral scanners
OCT: Optical coherence chromatography
